# Synergistic Use of Gold Nanoparticles (AuNPs) and “Capillary Enzyme-Linked Immunosorbent Assay (ELISA)” for High Sensitivity and Fast Assays

**DOI:** 10.3390/s18010055

**Published:** 2017-12-26

**Authors:** Wan-Joong Kim, Hyo Young Cho, Bongjin Jeong, Sangwon Byun, JaeDoo Huh, Young Jun Kim

**Affiliations:** 1 Medical-Device Lab, Electronics and Telecommunications Research Institute, Daejeon 305-700, Korea; kokwj@hanmail.net (W.-J.K.); deardol@etri.re.kr (H.Y.C.); jbj0919@etri.re.kr (B.J.); 2 Department of Electronics Engineering, Incheon National University, Incheon 22012, Korea; swbyun@inu.ac.kr; 3 Hyper-connected Basic Research Lab, Electronics and Telecommunications Research Institute, Daejeon 305-700, Korea; jdhuh@etri.re.kr

**Keywords:** capillary ELISA, ImmunoGold conjugate, C-reactive protein (CRP), point-of-care (POC)

## Abstract

Using gold nanoparticles (AuNPs) on “capillary enzyme-linked immunosorbent assay (ELISA)”, we produced highly sensitive and rapid assays, which are the major attributes for point-of-care applications. First, in order to understand the size effect of AuNPs, AuNPs of varying diameters (5 nm, 10 nm, 15 nm, 20 nm, 30 nm, and 50 nm) conjugated with Horseradish Peroxidase (HRP)-labeled anti-C reactive protein (antiCRP) (AuNP•antiCRP-HRP) were used for well-plate ELISA. AuNP of 10 nm produced the largest optical density, enabling detection of 0.1 ng/mL of CRP with only 30 s of incubation, in contrast to 10 ng/mL for the ELISA run in the absence of AuNP. Then, AuNP of 10 nm conjugated with antiCRP-HRP (AuNP•antiCRP-HRP) was used for “capillary ELISA” to detect as low as 0.1 ng/mL of CRP. Also, kinetic study on both 96-well plates and in a capillary tube using antiCRP-HRP or AuNP•antiCRP-HRP showed a synergistic effect between AuNP and the capillary system, in which the fastest assay was observed from the “AuNP capillary ELISA”, with its maximum absorbance reaching 2.5 min, while the slowest was the typical well-plate ELISA with its maximum absorbance reaching in 13.5 min.

## 1. Introduction

Recent interest in point-of-care (POC) applications [1] has prompted researchers to develop a wide range of immunoassay methods that display low-cost enhanced performance while not requiring help from an expert or facilities. A paper-based system [2,3] has been most widely utilized for POC purposes. However, limits in sensitivity are understood to be the major barrier for wider applications. Capillary-tube assay systems, in spite of their simple structure, have not been as successful for POC purposes. Although some capillary systems have been reported with emphasis on achieving a low limit of detection (LOD) [4,5,6], the single-step process [6,7,8] and multi-analyte detection [9,10], those capillary systems were mostly not as successful due either to LOD [4,5,7,8,10] or to complicated fabrication steps [6]. 

Recently, nanomaterials of gold [11,12], platinum [13], iron oxide (Fe_3_O_4_) [14], and graphene oxide [15] have drawn much attention in the field of immunoassay because those synthetic nano-catalysts [16] are stable and can be manufactured at low cost. In particular, AuNPs have been reported to be very efficient catalysts compared with other metal catalysts, including Pd, Ag, Pt and Cu [11]. For example, AuNPs conjugated with HRP-labeled antiCA15-3 were utilized for the sandwich immunoassay on a well-plate to produce enhanced performance [12]. 

Previously, we reported a highly-sensitive assay in which a capillary tube was used as a platform for POC purposes [5]. In ongoing research to develop a high-performance “capillary ELISA”, we hereby introduce the utilization of AuNPs into the capillary system in an effort to combine the catalytic effect of AuNP and the advantages of the capillary system. Based on typical surface chemistry in the simple structure of a capillary tube, the combined assay system indeed resulted in enhancement in sensitivity and assay time. CRP was used as a target analyte, since CRP is a useful biomarker for coronary artery disease [17] and inflammation [18]. Recently, development of an assay method to detect biomarkers in saliva has drawn increasing interest due to its non-invasive nature as [19,20]. However, because biomarker concentrations are usually much lower in saliva than in blood [21], highly sensitive detecting techniques are necessary. A combination of the “AuNP enzyme” (AuNP•antiCRP-HRP) and the “capillary ELISA”, which produces multiple benefits such as high sensitivity, short assay time and the shift of the linear region to lower concentration range, is likely to be an advantageous candidate as an assay system towards salivary CRP for POC purposes.

## 2. Experimental

### 2.1. Materials and Chemical Reagents

Gold nanoparticles (AuNPs) of different diameters (5 nm, 10 nm, 15 nm, 20 nm, 30 nm and 50 nm) were purchased from BBI Solution (Ted Pella, Redding, CA, USA). Capture antiC-reactive protein (antiCRP, 4C28-C5), detection antiCRP (4C28-CRP135), and C-reactive protein (CRP) antigen (8C72) were purchased from HyTest Ltd. (Turku, Finland). AntiCRP conjugated with HRP (antiCRP-HRP) was purchased from abcam^®^ (ab19175). Glutaraldehyde solution (GA, 25% in H_2_O, G5882), sodium cyanoborohydride (NaBH_3_CN), 3-aminopropyltriethoxysilane (APTES, 440140), 3,3′,5,5′-tetramethylbenzidine solution (TMB, T0440-100ML), human serum (from human male AB plasma, H4522-100ML) and bovine serum albumin (BSA, A7030-50G) were purchased from Sigma-Aldrich (Saint Louis, MO, USA). Blocker^TM^ Casein (37528) and 20x PBS Tween-20 (28352) were purchased from Thermo Fisher (Waltham Boston, MA, USA) and used in experiment as obtained without any dilution. Capillary tubes (cat. no. 1-000-0050, capacity 5 µL, length 32 mm, OD 0.95, and ID 0.4 mm) were purchased from Drummond Scientific Co. (Broomall, PA, USA). Well plates (96-well, polystyrene) were purchased from Thermo Fisher Scientific (Nunc, Waltham, MA USA). While the immunoassays performed in 96-well plates were measured by Infinite 200 PRO (TECAN Group Ltd., Hombrechtikon, Switzerland), a house-made miniaturized optical detection was used for analyzing “capillary ELISA”. 

### 2.2. Preparation of AuNPs Conjugated with antiCRP-HRP (AuNP•antiCRP-HRP)

AuNP•antiCRP-HRPs were prepared using AuNPs of different diameters (5 nm, 10 nm, 15 nm, 20 nm, 30 nm and 50 nm) based on procedures previously described [22]. Briefly, using AuNP of 10 nm as an example, 1.0 mL of the AuNP solutions (9.46 nM) was mixed with 20 μL of potassium carbonate (K_2_CO_3_, 40 mM) by adjusting pH at 9. The reaction mixture was shaken for 1 min followed by adding 100 μL of antiCRP-HRP (100 μg/mL). Again, the solution mixture was shaken for 5 more minutes, added with 100 μL of 10% BSA in DI water and incubated for 1 h. In order to remove the unbound antibodies and BSA, the solutions were centrifuged at 12,000 rpm for 20 min. AuNP•antiCRP-HRPs were collected and dissolved in 1 mL of 50 mM PBS containing 0.1% NaN_3_ by keeping the pH at 8.0. The concentration of AuNP•antiCRP-HRPs was maintained to be the same as the AuNPs as obtained from BBI, which is 9.46 nM for 10 nm. The AuNP•antiCRP-HRPs solution was used in well-plate and capillary ELISAs as had been prepared. 

### 2.3. Immobilization of the Capture antiCRP Inside a Capillary Tube

Immobilization of the captured antiCRP on the inner surface of a capillary tube was processed via vapor-phase amination by following the method reported previously [5]. Briefly, a glass capillary tube was treated with oxygen plasma for 300 s (30 Pa, 100 mL/min and 100 watts). APTES (2 mL) in a round-bottom flask was heated up to 120 °C for 20 min to be carried over to micro-capillary tubes. The capillary tubes were then imbued with solution mixture of glutaraldehyde (aqueous 25 wt %) and sodium cyanoborohydride (NaBH_3_CN) (1.0 wt % to the total reaction mixture) followed by incubation at room temperature for 4 h. After washing with deionized water a few times, the aldehyde-functionalized capillary tubes were reacted with 5 μL of antiCRP (4C28-C8, 10 μg/mL) and incubated overnight at 4 °C. The capillary tubes were washed with 0.01% tween 20 (1x PBS, pH 7.4), imbued with 5 μL of casein, incubated for 30 min, and again washed with 0.01% tween 20 (1x PBS, pH 7.4).

### 2.4. Immunoassays on a 96 Well-Plate

The well-plate ELISA was processed using antiCRP-HRP and AuNP•antiCRP-HRP as detection antibody. Into each well of a 96-well plate, 50 μL of antiCRP (10 μg/mL) was added followed by incubation at 4 °C overnight. Each well was washed with 0.01% tween 20 (1x PBS, pH 7.4) followed by incubation with 200 μL of casein for 30 min. After washing the well plate with 0.01% tween 20 (1x PBS, pH 7.4), predetermined amount of CRPs (0.1, 0.5, 1, 5, 10, 100, and 1000 ng/mL) was added into each well in two series. For control, only buffer solution in the absence of CRP was added followed by 30 min of incubation. After washing the well plate with 0.01% tween 20 (1x PBS, pH 7.4), one array of the well was combined with 50 μL of antiCRP-HRP and the other array was combined with AuNP•antiCRP-HRP followed by 30 min of incubation. After washing the plate with 0.01% tween 20 (1x PBS, pH 7.4), each well was combined with 50 μL of TMB solution. The optical density of each ELISA was measured 60 s after TMB solution was added by reading at 650 nm on the Tecan reader. 

### 2.5. Home-Made Optical Detector

In order to analyze capillary immunoassays, a miniaturized homemade detector was used [5], which had been constructed based on a photo-interrupter (GP1S092HCPIF, Sharp, Osaka, Japan). The intensity of emitting light (LD) was controlled by varying input current using pulse width modulation (PWM). The LD was made of gallium arsenide (GaAs) with maximum emitting wavelength at 950 nm and photodiode (PD) was made of silicon (Si) with maximum sensitivity wavelength at 930 nm. Optical density of the “capillary ELISA” was processed by measuring transmittance via reading voltage values on the detector (Figure S1). The output voltage from the phototransistor was amplified by an operation amplifier and then converted by a 10-bit analog-to-digital converter (ADC) to represent the voltage by values ranging from 0 to 1000.

### 2.6. Immunoassays in Capillary Tubes

The capillary tubes, which had been immobilized with captured antiCRP, were imbued with different concentrations of CRP (0.1, 0.5, 1, 5, 10, 100, and 1000 ng/mL), which had been prepared both in PBS buffer and serum. For the control tests, PBS solutions or serum were infused into the capillary tubes in the absence of CRP. After 30 min of incubation the capillary tubes were washed with 0.01% tween 20 (1x PBS, pH 7.4). The capillary tubes were now infused with AuNPs• antiCRP-HRP followed by 30 min of incubation. After washing with 0.01% tween 20 (1x PBS, pH 7.4) the capillary tubes were infused with 50 µL of TMB solution. Optical density was measured by reading voltage values on a homemade detector 30 s after addition of TMB solution. When monitoring experiments were carried out, change of optical density was followed for 20 min by collecting read-out values every 30 s on a homemade detector.

### 2.7. SEM Analysis of the “AuNP Capillary ELISA”

The analysis of the capillary ELISA involving AuNP•antiCRP-HRP was also confirmed with field emission scanning electron microscopy (FE-SEM, FEI siron-400 FE-SEM) by counting the number of AuNPs participated in the assay within the area of 1 µm^2^. In order to prepare samples for the SEM images, the capillary tubes that had undergone “capillary ELISA” based on AuNP•antiCRP-HRP were broken into small pieces and coated with platinum in 10 nm thickness. The number of gold nanoparticles on the capillary surface was obtained by counting the nanoparticles directly from the SEM images.

## 3. Results and Discussion

### 3.1. Size Effect of AuNPs on ELISA

**Figure 1:**
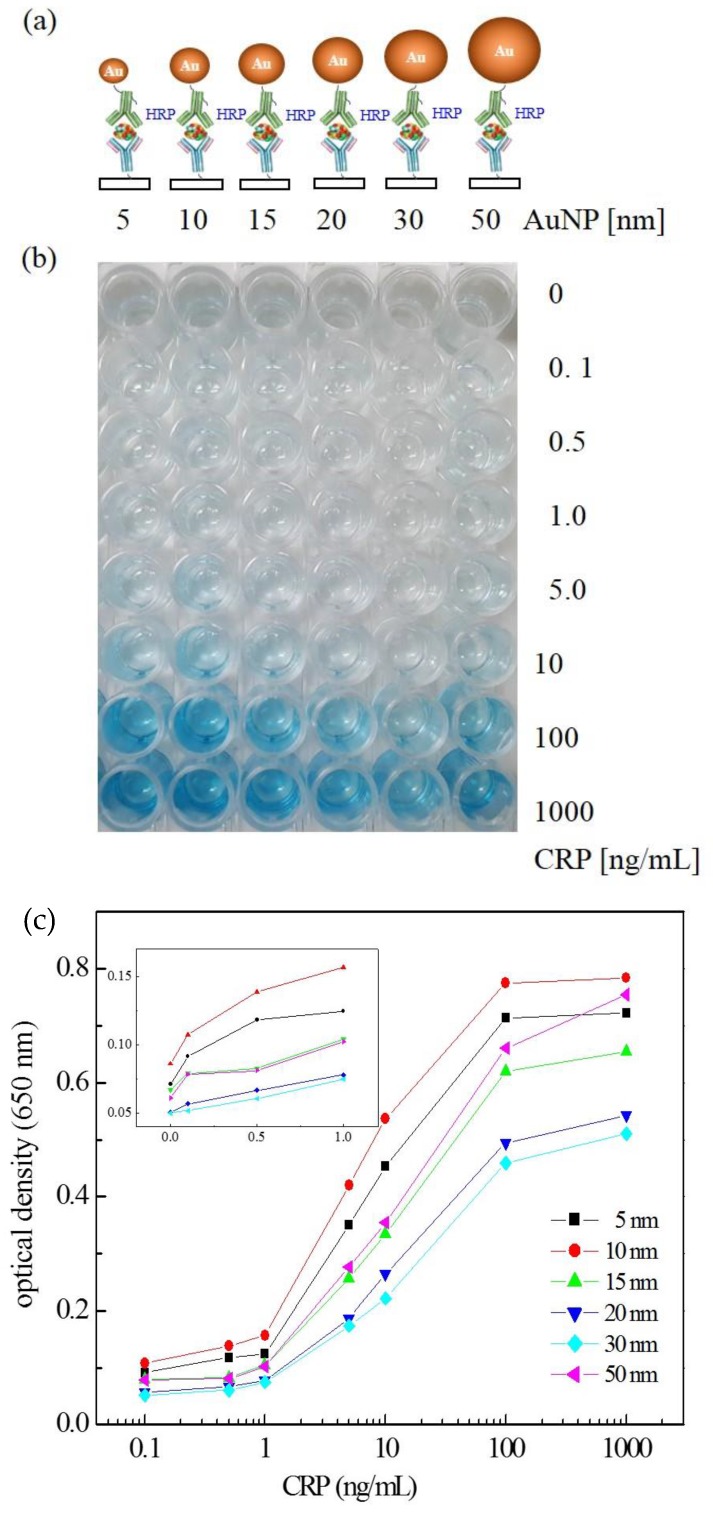
(**a**) The schematic view of the sandwich ELISA using AuNP•antiCRP-HRP with varying diameters of the AuNPs involved, (**b**) the picture of 96-well plate of the resulting ELISA produced based on the AuNP•antiCRP-HRPs, and (**c**) the plot of optical density and CRP concentration for the “AuNP ELISA” with varying diameter of AuNPs.

**Figure 2:**
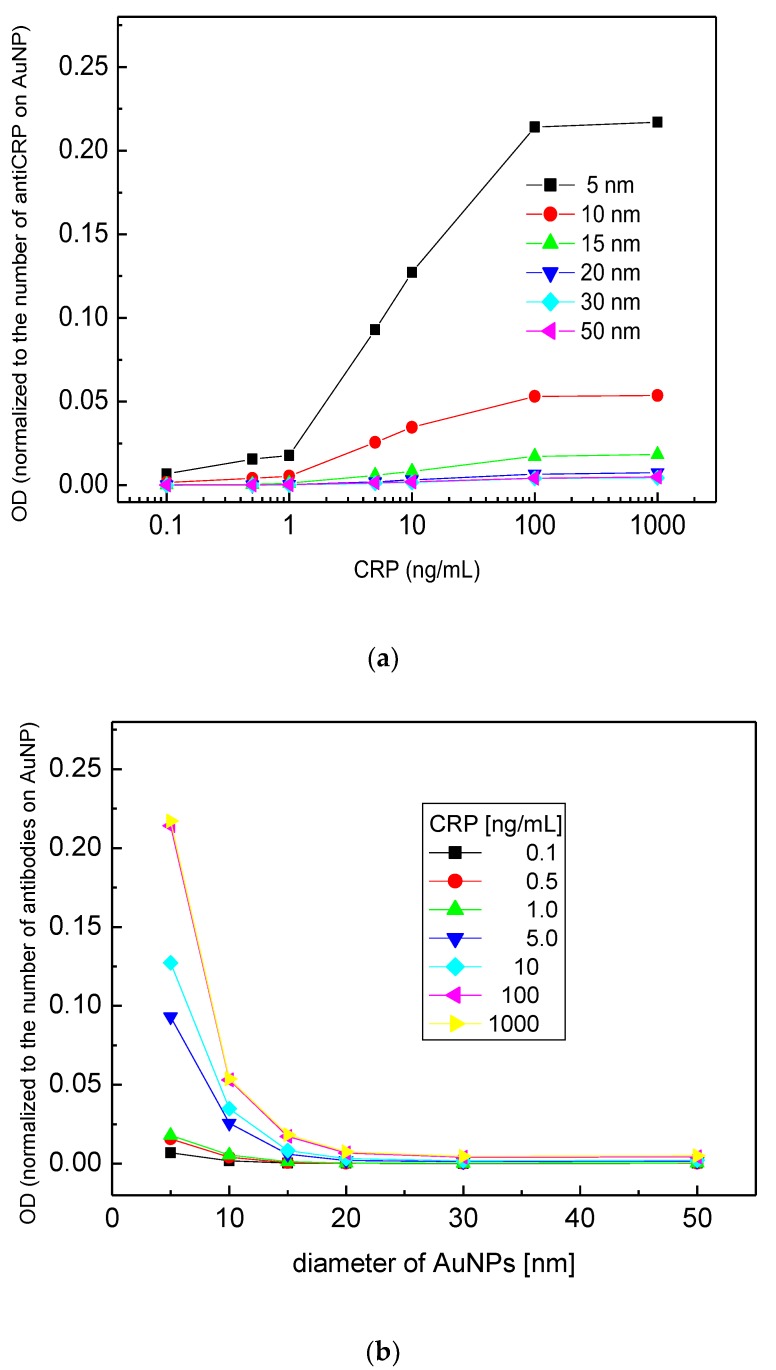
Normalized plots of Figure 1c based on encapsulating antibodies of each AuNP when optical densities were plotted against (**a**) CRP concentration, and (**b**) diameters of AuNPs.

**Figure 3:**
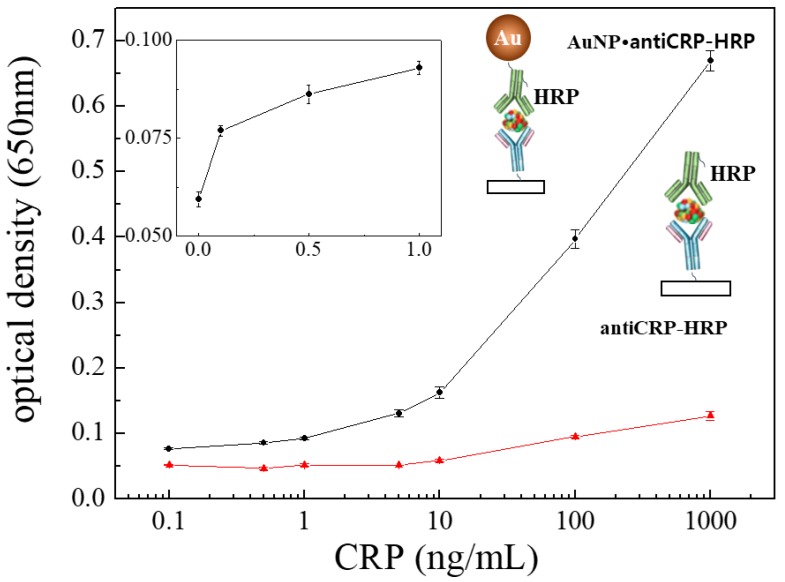
Logarithmic plots of optical density against CRP concentration of the “AuNP ELISA” (AuNP 10 nm) (black line) and the conventional ELISA (red line) with the inset representing the linear plot of the lower concentration range of the “AuNP ELISA”.

A new assay system was devised by applying AuNP•antiCRP-HRP on the “capillary ELISA” based on previous reports about enhanced performance of the AuNP conjugated with HRP-labeled antibody (AuNP•Ab-HRP) [12] and of the “capillary ELISA” [5]. First, in order to study the size effect of AuNP, a series of AuNP•antiCRP-HRPs were prepared using different diameters of AuNP (5 nm, 10 nm, 15 nm, 20 nm, 30 nm, and 50 nm) and sandwich-type immunoassays were performed on a 96-well plate. Figure 1a shows the schematic view of the different AuNP•antiCRP-HRPs in complex with CRP and captured anti-CRP. Figure 1b well shows the characteristic blue color of the assay product that formed 30 s after adding TMB substrate solution to the sandwich complex. The characteristic blue color with maximum absorption at around 650 nm has been caused by oxidation of TMB by H_2_O_2_ catalyzed by HRP. Figure 1c is the plot of CRP concentrations used for the assay against the resulting optical densities with the inset representing a linear plot at lower concentration range. It is surprising that with only 30 s of incubation time at 0.1 ng/mL was recognized for most of the AuNPs involved, which is in stark contrast with the 20 min to 30 min recognized for the normal ELISA. Such shortening of assay time is a valuable asset for POC applications. However, the optical densities do not seem to have exact correlation with the AuNP size, with the largest optical value for AuNP of 10 nm and the smallest for 30 nm, which is in apparent disagreement with the previous report of inverse relationship of AuNP size with catalytic effect [23]. In Figure 2a, the plots of CRP concentration against optical density were represented by normalizing the optical values with the number of antiCRP-HRPs conjugated on AuNPs. The number of antiCRP-HRP on different AuNPs was determined to be 3, 13, 32, 66, 102 and 142 for AuNPs of 5 nm, 10 nm, 15 nm, 20 nm, 30 nm and 50 nm respectively based on the calculation of IgGs adsorbed on AuNPs [24]. Now, the inverse relationship of the optical density with the diameter of AuNP is clear. In order better to see the size effect on catalytic efficiency, the normalized values of the optical densities were plotted against diameters of AuNPs (Figure 2b), in which the inverse relationship plateaued with AuNPs larger than 20 nm. We reported similar results explaining that the HRP activity was much weakened for AuNPs larger than 15 nm [25]. Our results show that even as the number of HRPs increases, the catalytic efficiency decreases, suggesting the catalytic effect of size is a dominant factor in deciding peroxide-like activity. While the apparent catalytic efficiency is maximum for AuNP of 10 nm (Figure 1c), the normalized catalytic efficiency is highest for the smallest AuNP of 5 nm, which is thought to suggest some synergistic catalytic effect is occurring between particle size and number of HRPs conjugated on AuNPs. Figure 3 shows the plots of optical density against CRP concentration corresponding to AuNP ELISA (10 nm) and conventional ELISA. In each ELISA, 30 s of incubation was used after adding TMB solution. It was surprising to discover that only 30 s of incubation time was enough to recognize 0.1 ng/mL of CRP for ELISA with AuNP•antiCRP-HRP as shown in the inset of Figure 3, while 10 ng/mL of CRP seemed to have been barely recognizable for the typical ELISA.

### 3.2. “Capillary ELISA” Using AuNP•antiCRP-HRP

**Figure 4:**
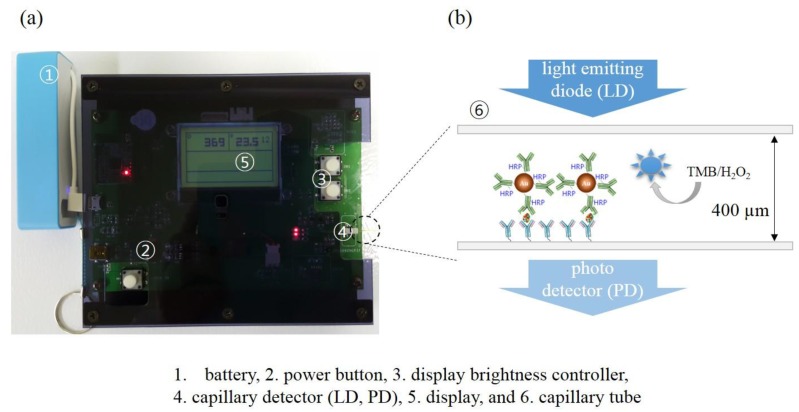
(**a**) The picture of the manufacture optical reader, and (**b**) schematic view illustrating the “AuNP capillary ELISA”.

**Figure 5:**
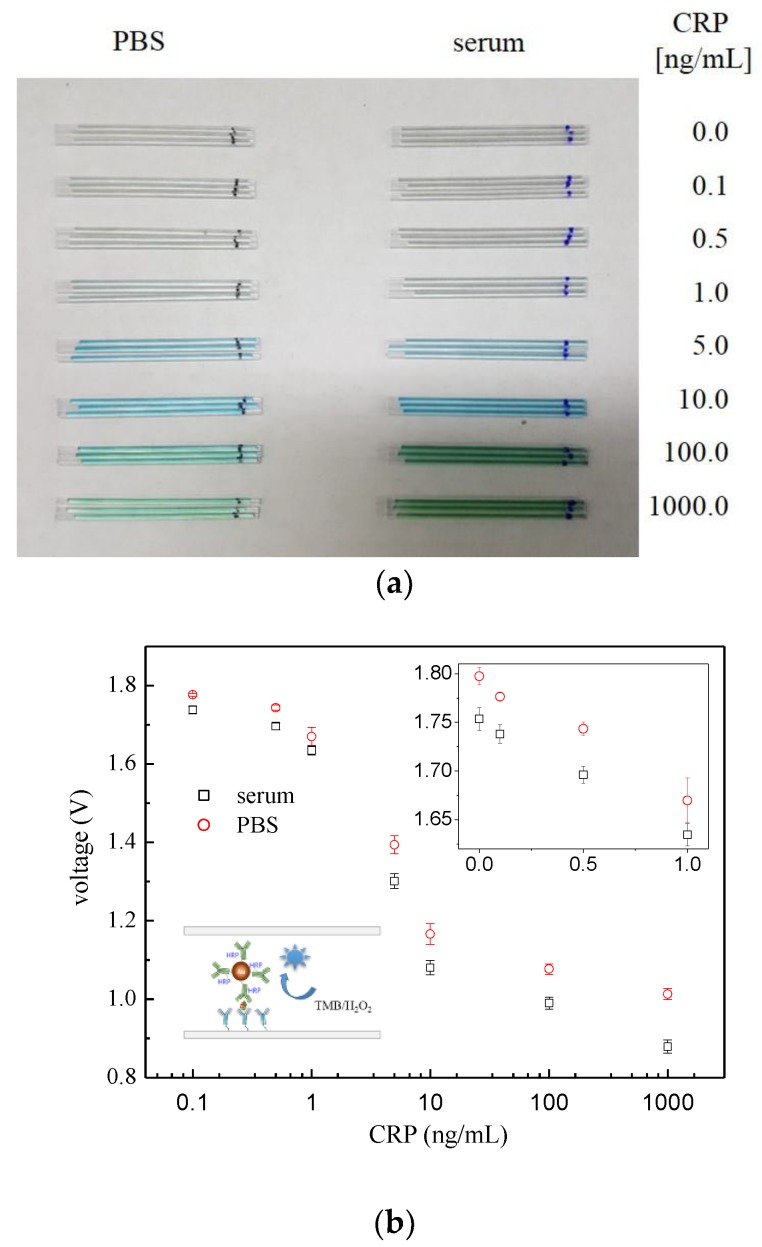
(**a**) Picture of the capillary tubes that had undergone “AuNP capillary ELISA” either under PBS or serum, and (**b**) their logarithmic plot of optical density expressed in voltage against CRP concentration with the inset representing linear plot in the lower concentration range.

**Figure 6:**
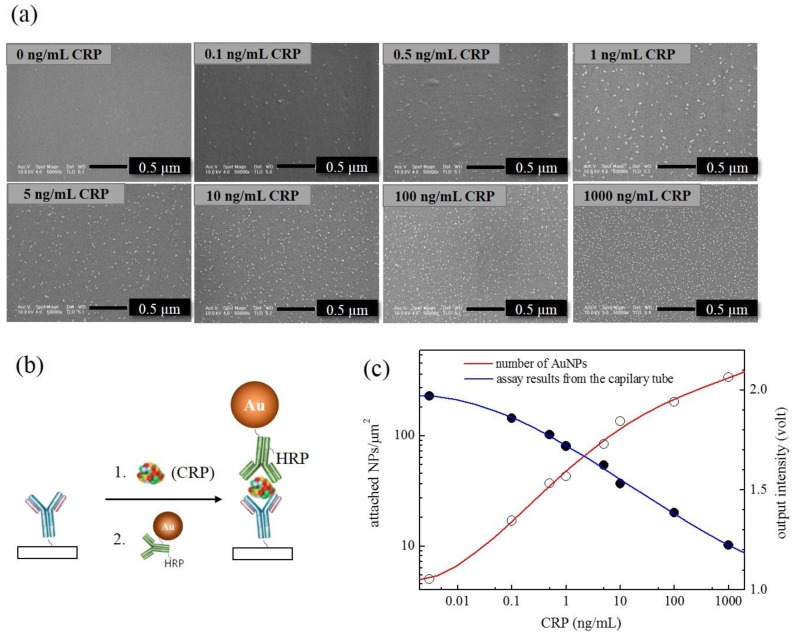
(**a**) Images of the FE-SEM produced from the pieces of the capillary tubes that had undergone “AuNP capillary ELISA” with varying amount of CRP, (**b**) schematic representation of the “AuNP ELISA”, and (**c**) the plots obtained from the “AuNP capillary ELISA” based on optical density (blue line) and number of AuNPs involved (red line).

**Figure 7:**
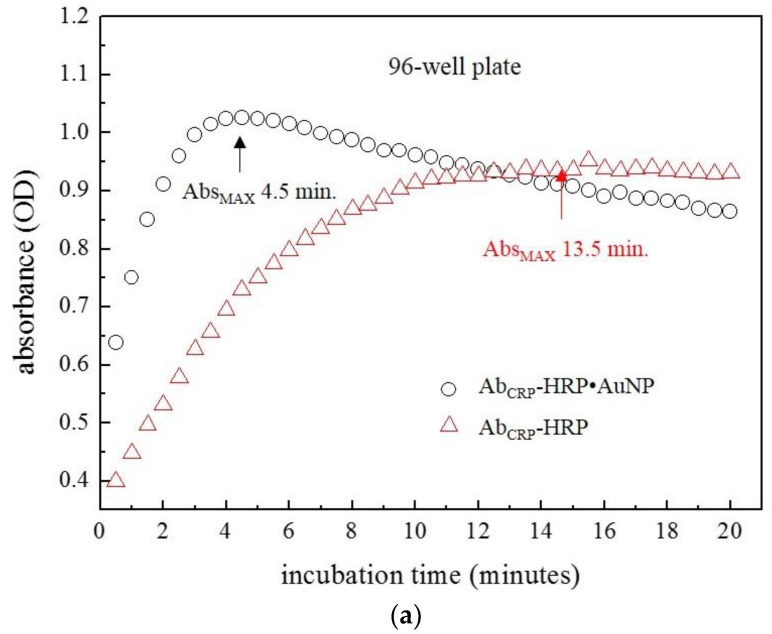

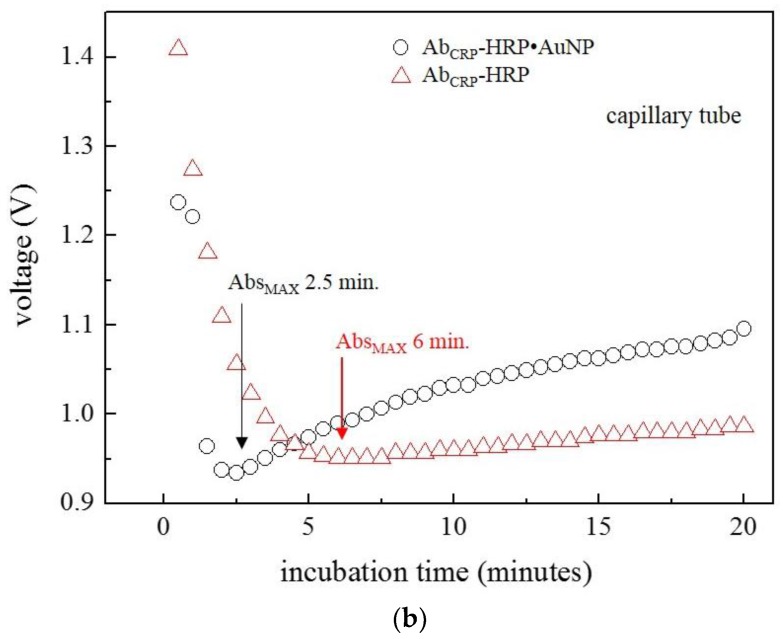
Plots representing the monitoring of the ELISAs either on (**a**) the 96-well plate, or (**b**) the capillary tube using AuNP•antiCRP-HRP (black circle) and antiCRP-HRP (red triangle) as indicator antibodies.

Now, choosing the AuNP•antiCRP-HRP of 10 nm, which had shown the largest apparent optical density, a series of ELISAs were carried out in capillary tubes. Figure 4a shows the optical detector that had been manufactured for measuring “capillary ELISAs” together with the schematic description of the sandwich-type AuNP ELISA occurring inside the tube (Figure 4b). The “AuNP capillary ELISAs” were processed either in PBS or serum by varying the concentration of CRP from 0.1 ng/mL to 1000 ng/mL including the control test. Figure 5a is the picture for the capillary tubes that had undergone “AuNP capillary ELISA”, in which deepening of the characteristic blue color was observable as the CRP concentration increased. The “AuNP capillary ELISA” showed not only high sensitivity of detecting 0.1 ng/mL of CRP but also shift of the linear region to lower concentration range. At least the high sensitivity is not ascribed to the homemade optical detector as had been confirmed in the previous report, in which the homemade detector produced almost the same detection capability as the ELISA reader [5]. When compared with “AuNP well-plate ELISA”, the shift of the dynamic range to lower concentration can be seen for the capillary system, while the sensitivity of the “AuNP capillary ELISA” seemed to be around same level. Also, the results of the “AuNP capillary ELISA” were confirmed by analyzing the capillary tubes that had undergone “AuNP ELISA” under FE-SEM (Figure 6a). Since each sandwich-type ELISA complex contains one AuNP (Figure 6b), the assay can also be studied by counting the number of AuNP involved. The plot of AuNP counting turned out to be in mirror image with that of the optical density value (Figure 7c), which confirms the validity of the “AuNP capillary ELISA”.

In order to understand the kinetics of the “capillary ELISA”, color change was followed by measuring incubation time upon addition of TMB to sandwich-type complex. Well-plate ELISA (Figure 7a) and “capillary ELISA” (Figure 7b) were carried out the using either AuNP•antiCRP-HRP or antiCRP-HRP as a “indicator antibody”. In both the well-plate and the capillary tube, faster assays were observed when AuNPs were involved, which is thought to have been contributed by the catalytic effect of AuNPs. Also, the capillary system resulted in a faster assay than the well-plate ELISA, which may have been caused by short diffusion length in the capillary tube. The catalytic effect of AuNP and short diffusion length provided by the capillary tube seemed have contributed faster kinetics resulting in rapid assay. 

## 4. Conclusions

The use of AuNP in the “capillary ELISA” synergistically resulted in high sensitivity and fast assay time. In measuring the size effect of AuNP on ELISA, the inverse relationship between AuNP diameters with sensitivity was observed, especially when optical density was normalized by the number of antiCRP-HRP encapsulating each AuNP. When only 30 s of incubation time after adding TMB substrate was used, 0.1 ng/mL CRP was detected for the “AuNP well-plate ELISA”. When AuNPs were used in the “capillary ELISA”, dynamic range in the low-concentration range was observed together with high sensitivity. Kinetic studies of both the well-plate and capillary ELISAs supported synergistic effects of AuNP and capillary systems on shortened assay time. Overall the synergistic utilization of AuNP and capillary system produced high sensitivity, dynamic range in the low concentration range and fast kinetics, which are significant attributes for POC applications. 

## Supplementary Materials

The following are available online at http://www.mdpi.com/1424-8220/18/1/55/s1.

## References

[1] Sun J., Xianyu Y., Jiang X. (2014). Point-of-care biochemical assays using gold nanoparticle-implemented microfluidics. Chem. Soc. Rev..

[2] Hu J., Wang S., Wang L., Li F., Pingguan-Murphy B., Lu T.J., Xu F. (2014). Advances in paper-based point-of-care diagnostics. Biosens. Bioelectron..

[3] Cate D.M., Adkins J.A., Mettakoonpitak J., Henry C.S. (2015). Recent developments in paper-based microfluidic devices. Anal. Chem..

[4] Funano S., Henares T.G., Kurata M., Sueyoshi K., Endo T., Hisamoto H. (2013). Capillary-based enzyme-linked immunosorbent assay for highly sensitive detection of thrombin-cleaved osteopontin in plasma. Anal. Biochem..

[5] Kim W.-J., Hyun S.H., Cho H.Y., Byun S., Kim B.K., Huh C., Chung K.H., Kim Y.J. (2016). Sensitive “capillary elisa” via vapor-phase surface modification. Sens. Actuators B Chem..

[6] Mohammed M.I., Desmulliez M.P.Y. (2014). Autonomous capillary microfluidic system with embedded optics for improved troponin I cardiac biomarker detection. Biosens. Bioelectron..

[7] Funano S.-I., Sugahara M., Henares T.G., Sueyoshi K., Endo T., Hisamoto H. (2015). A single-step enzyme immunoassay capillary sensor composed of functional multilayer coatings for the diagnosis of marker proteins. Analyst.

[8] Wakayama H., Henares T.G., Jigawa K., Funano S.-I., Sueyoshi K., Endo T., Hisamoto H. (2013). Design of a single-step immunoassay principle based on the combination of an enzyme-labeled antibody release coating and a hydrogel copolymerized with a fluorescent enzyme substrate in a microfluidic capillary device. Lab Chip.

[9] Henares T.G., Shirai A., Sueyoshi K., Endo T., Hisamoto H. (2015). Fabrication and packaging of a mass-producible capillary-assembled microchip for simple and multiplexed bioassay. Sens. Actuators B Chem..

[10] Edwards A.D., Reis N.M., Slater N.K.H., Mackley M.R. (2011). A simple device for multiplex ELISA made from melt-extruded plastic microcapillary film. Lab Chip.

[11] Comotti M., Della Pina C., Matarrese R., Rossi M. (2004). The catalytic activity of “naked” gold particles. Angew. Chem. Int. Ed..

[12] Ambrosi A., Airò F., Merkoçi A. (2010). Enhanced gold nanoparticle based ELISA for a breast cancer biomarker. Anal. Chem..

[13] Li W., Chen B., Zhang H., Sun Y., Wang J., Zhang J., Fu Y. (2015). BSA-stabilized Pt nanozyme for peroxidase mimetics and its application on colorimetric detection of mercury(II) ions. Biosens. Bioelectron..

[14] Gao L., Zhuang J., Nie L., Zhang J., Zhang Y., Gu N., Wang T., Feng J., Yang D., Perrett S. (2007). Intrinsic peroxidase-like activity of ferromagnetic nanoparticles. Nat. Nanotechnol..

[15] Song Y., Qu K., Zhao C., Ren J., Qu X. (2010). Graphene oxide: Intrinsic peroxidase catalytic activity and its application to glucose detection. Adv. Mater..

[16] Wei H., Wang E. (2013). Nanomaterials with enzyme-like characteristics (nanozymes): Next-generation artificial enzymes. Chem. Soc. Rev..

[17] Silva D., Pais de Lacerda A. (2012). High-sensitivity C-reactive protein as a biomarker of risk in coronary artery disease. Rev. Port. Cardiol..

[18] Yeh E.T.H., Willerson J.T. (2003). Coming of age of C-reactive protein. Using Inflamm. Mark. Cardiol..

[19] Mishra S., Saadat D., Kwon O., Lee Y., Choi W.-S., Kim J.-H., Yeo W.-H. (2016). Recent advances in salivary cancer diagnostics enabled by biosensors and bioelectronics. Biosens. Bioelectron..

[20] Yoshizawa J.M., Schafer C.A., Schafer J.J., Farrell J.J., Paster B.J., Wong D.T.W. (2013). Salivary biomarkers: Toward future clinical and diagnostic utilities. Clin. Microbiol. Rev..

[21] Punyadeera C., Dimeski G., Kostner K., Beyerlein P., Cooper-White J. (2011). One-step homogeneous C-reactive protein assay for saliva. J. Immunol. Methods.

[22] Kim W.-J., Cho H.Y., Kim B.K., Huh C., Chung K.H., Ahn C.-G., Kim Y.J., Kim A. (2015). Highly sensitive detection of cardiac troponin I in human serum using gold nanoparticle-based enhanced sandwich immunoassay. Sens. Actuators B Chem..

[23] Fenger R., Fertitta E., Kirmse H., Thunemann A.F., Rademann K. (2012). Size dependent catalysis with CTAB-stabilized gold nanoparticles. Phys. Chem. Chem. Phys..

[24] Bell N.C., Minelli C., Shard A.G. (2013). Quantitation of IgG protein adsorption to gold nanoparticles using particle size measurement. Anal. Methods.

[25] Wu H., Liu Y., Li M., Chong Y., Zeng M., Lo Y.M., Yin J.-J. (2015). Size-dependent tuning of horseradish peroxidase bioreactivity by gold nanoparticles. Nanoscale.

