# Supplementary material for: Synergistic Use of Gold Nanoparticles (AuNPs) and “Capillary Enzyme-Linked Immunosorbent Assay (ELISA)” for High Sensitivity and Fast Assays
Source: Sensors (Basel). 2017 Dec 26;18(1):55. doi: 10.3390/s18010055 (PMC5795359; doi:10.3390/s18010055)

Synergistic use of gold nanoparticle (AuNP) and "capillary enzyme-linked immunosorbent assay (ELISA)" for high sensitivity and fast assay.

Wan-Joong Kim, Hyo Young Cho, Bongjin Jeong, Sangwon Byun, JaeDu Huh, and Young Jun Kim

Supporting information 1.

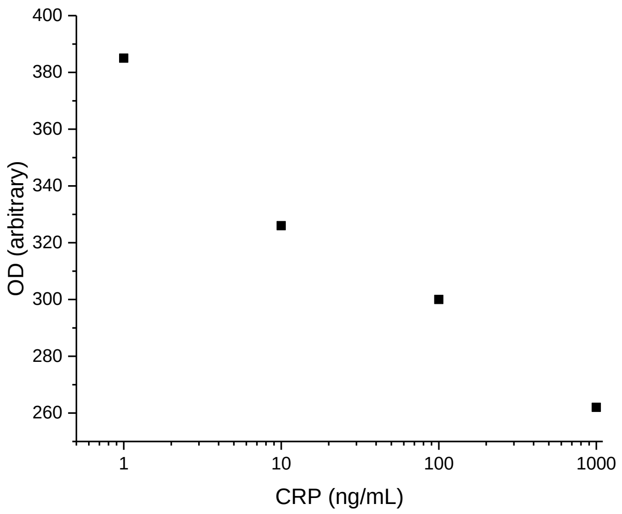

(**a**)

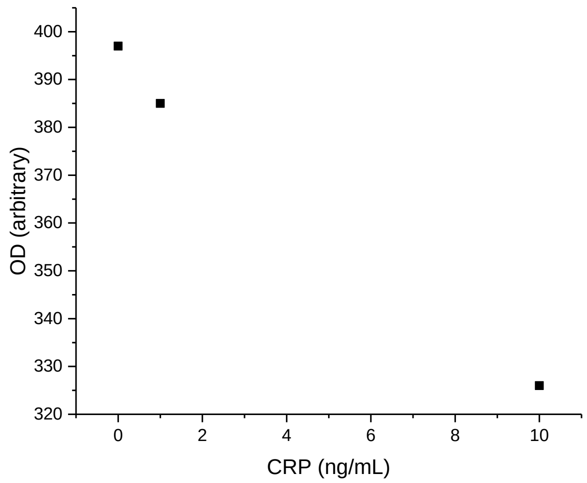

(**b**)

Figure S1. Plots of CRP and optical density carried out in "capillary ELISA" using artificial saliva as assay medium in which (**a**) is in logarithmic scale and (**b**) in linear scale including 0 ng/mL of CRP.

Supporting information 2.

Table S1. The information about AuNP size.

Size of Gold Nanoparticle	Mean size Particle Size	Maximum Acceptable %CV	Number of Odd Shapes per 100 Particles
5 nm	4.5–6.0 nm	15%	≤5
10 nm	9.0–11.0 nm	10%	≤5
15 nm	14.0–16.5 nm	10%	≤5
20 nm	19.0–21.0 nm	8%	≤5
30 nm	28.0–32.0 nm	8%	≤5

Supporting information 3.

In the plot of the Figure 5, the concentration of CRP for serum is apparent values that did not consider the background concentration of CRP in serum. The background concentration of CRP in serum can be calculated to be around 0.5 ng/mL. So the actual concentrations of CRP used in Figure 5 are 0.1 ng/mL, 0.6 ng/mL, 1.5ng/mL, 10 ng/mL and 100.5 ng/mL and 1000.5 ng/mL.

Supporting Information 4.

Reference: Erickson, H.P. Size and Shape of Protein Molecules at the Nanometer Level Determined by Sedimentation, Gel Filtration, and Electron Microscopy. *Biol. Proced. Online* **2009**, *11*, doi:10.1007/s12575-009-9008-x.

All proteins have approximately the same density 1.37 g/cm^3. The partial specific volume is the reciprocal of the density. So the partial specific volume of proteins is 0.73 cm^3/g. The volume (V) occupied by a protein mass M in Dalton can be calculated as follows.

$$V\ (\text{nm}^3) = \frac{(0.73\ \text{cm}^3/\text{g}) \times (10^{21}\ \text{nm}^3/\text{cm}^3)}{6.02 \times 10^{23}\ \text{Da/g}} \times M\ (\text{Da})$$

Since, molecular weight of IgG is around 150 kDa and Molecular weight of HRP = 44 kDa.

$$\text{The calculated volume of IgG} = \frac{(0.73\ \text{cm}^3/\text{g}) \times (10^{21}\ \text{nm}^3/\text{cm}^3)}{6.02 \times 10^{23}\ \text{Da/g}} \times 1.5 \times 10^5\ (\text{Da}) = 1.82 \times 10^2\ \text{nm}$$

$$\text{The calculated volume of HRP} = \frac{(0.73\ \text{cm}^3/\text{g}) \times (10^{21}\ \text{nm}^3/\text{cm}^3)}{6.02 \times 10^{23}\ \text{Da/g}} \times 4.4 \times 10^4\ (\text{Da}) = 5.3 \times 10^1\ \text{nm}$$

Since the molecular weight of IgG and antiCRP is almost the same, the calculated molecular weight of antiCRP-HRP is approximated to be 150 + 44 [KDa] or 194 KDa

$$\text{Then the calculated volume of antiCRP-HRP} = \frac{(0.73\ \text{cm}^3/\text{g}) \times (10^{21}\ \text{nm}^3/\text{cm}^3)}{6.02 \times 10^{23}\ \text{Da/g}} \times 1.94 \times 10^5\ (\text{Da}) = 2.35 \times 10^2\ \text{nm}$$

and the volume ratio of antiCRP-HRP/IgG = 2.35/1.82~1.30. The actual number of antiCRP-HRP on AuNP can be approximated with the ratio values, 1.30.

size of AuNP [nm]	5	10	15	20	30	50
# of IgG on AuNP [23]	3	13	32	66	102	142
# of antiCRP-HRP on AuNP	2.3	10.0	24.6	50.8	78.5	109.2

Below is the plot redrawn by taking into account of the volume effect of HRP. Somewhat higher activity was shown compared with Figure 2a.

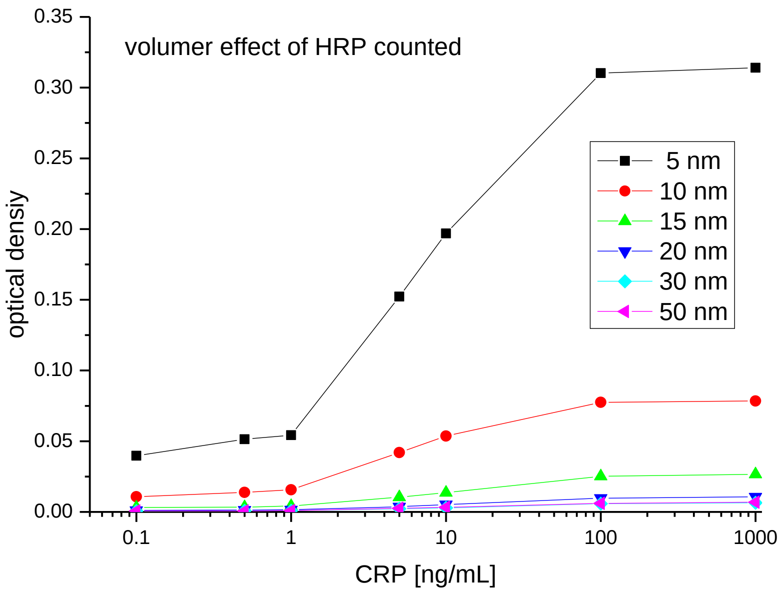

Supporting Information 5.

Pictures showing monitoring of the "capillary ELISA"

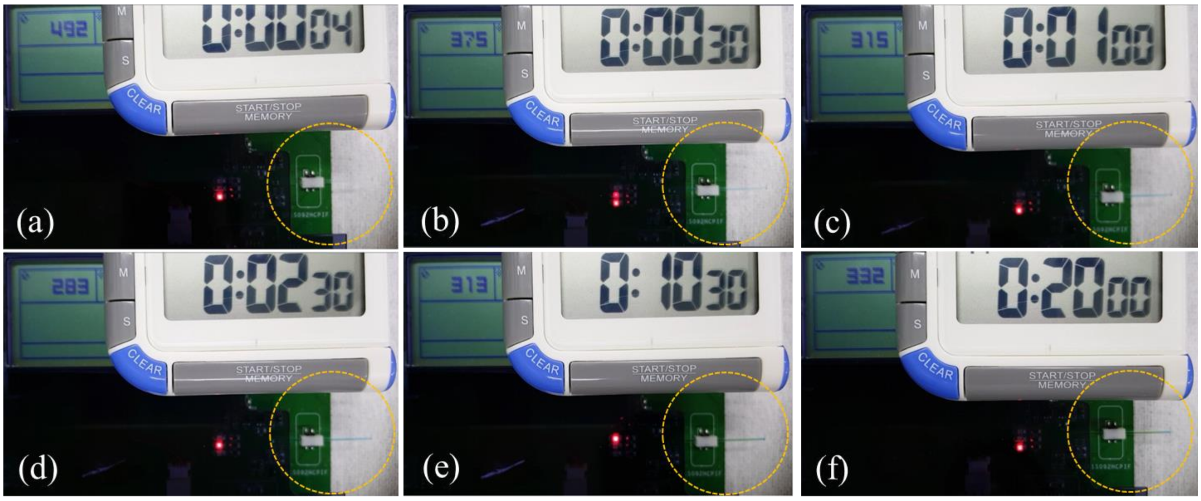

Supporting Information 6: Relative Activity of AuNP·antiCRP-HRP and anti-CRP-HRP

The plot of relative activity against CRP concentration has been obtained based on the data from figure 3. CRP concentrations corresponding to 0.1 ng/mL, 1.0 ng/mL, 10 ng/mL, 100 ng/mL and 1000 ng/mL were chosen for the plot.

relative activity = actual optical density from AuNP·antiCRP-HRP/actual optical density from anti-CRP-HRP

actual optical density = apparent optical density – optical density at 0 ng/mL of CRP

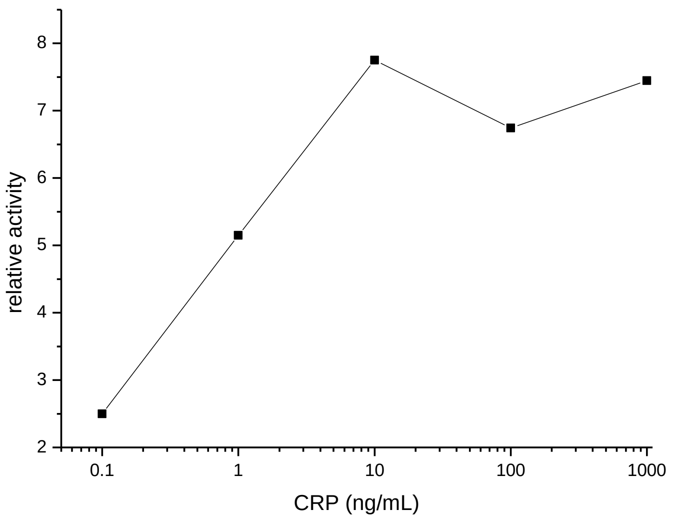

Supplement: Supplementary file 1 [file sensors-18-00055-s001.pdf]
